# The quantitative changes in the yeast Hsp70 and Hsp90 interactomes upon DNA damage

**DOI:** 10.1016/j.dib.2014.10.006

**Published:** 2014-11-07

**Authors:** Andrew W. Truman, Kolbrun Kristjansdottir, Donald Wolfgeher, Natalia Ricco, Anoop Mayampurath, Samuel L. Volchenboum, Josep Clotet, Stephen J. Kron

**Affiliations:** a Department of Molecular Genetics and Cell Biology, The University of Chicago, Chicago, IL 60637, USA; b Department of Biomedical Sciences, Midwestern University, Downers Grove, IL 60515, USA; c Departament de Ciències Bàsiques, Universitat Internacional de Catalunya, Barcelona, Catalunya, Spain; d Computation Institute, The University of Chicago, Chicago, IL 60637, USA; e Department of Pediatrics, The University of Chicago, Chicago, IL 60637, USA

## Abstract

The molecular chaperones Hsp70 and Hsp90 participate in many important cellular processes, including how cells respond to DNA damage. Here we show the results of applied quantitative affinity-purification mass spectrometry (AP-MS) proteomics to understand the protein network through which Hsp70 and Hsp90 exert their effects on the DNA damage response (DDR). We characterized the interactomes of the yeast Hsp70 isoform Ssa1 and Hsp90 isoform Hsp82 before and after exposure to methyl methanesulfonate. We identified 256 chaperone interactors, 146 of which are novel. Although the majority of chaperone interaction remained constant under DNA damage, 5 proteins (Coq5, Ast1, Cys3, Ydr210c and Rnr4) increased in interaction with Ssa1 and/or Hsp82. This data presented here are related to [1] (Truman et al., in press).

The mass spectrometry proteomics data have been deposited to the ProteomeXchange Consortium (http://proteomecentral.proteomexchange.org) via the PRIDE partner repository (Vizcaino et al. (2013) [2]) with the dataset identifier PXD001284.

## Specifications table

**Table:** 

Subject area	*Biology*
More specific subject area	*Molecular chaperones, Mass spectrometry, DNA damage response*
Type of data	^*18*^*O Quant LC–MS/MS Mass spectrometry data*
How data was acquired	*Mass spectrometry. Thermo LTQ Orbitrap XL*
Data format	*.Raw
Experimental factors	*Yeast cells expressing HIS*_*6*_*-tagged Ssa1 or Hsp82 were untreated or treated with 0.02% MMS for 3 h*
Experimental features	*HIS*_*6*_*-Ssa1 or HIS*_*6*_*-Hsp82 complexes were purified by IMAC and processed by mass spectrometry*
Data source location	*The University of Chicago, Chicago, IL, USA*
Data accessibility	Data are available with this article.
	The mass spectrometry proteomics data have been deposited to the ProteomeXchange Consortium (http://proteomecentral.proteomexchange.org) via the PRIDE partner repository with the dataset identifier PXD001284.

## Value of the data

• This data provides a comprehensive interactome of yeast Hsp70 (Ssa1).
• This data provides a comprehensive interactome of yeast Hsp90 (Hsp82).
• Offers new chaperone interactors that might be exploited in disease research.

## 1 Experimental design, materials and methods

100 ml of SKY4364 [3] were grown to an OD_600_ of 0.5 in YPD media. Cells were split into two flasks, one untreated and one which was subjected to 0.02% MMS for 3 h. Cells were harvested and HIS_6_-tagged Ssa1 along with the associated interactome was isolated as follows: Protein was extracted via bead beating in 500 µl Binding/Wash Buffer (50 mM Na-phosphate pH 8.0, 300 mM NaCl, 0.01% Tween-20). 200 µg of protein extract was incubated with 50 µl His-Tag Dynabeads (Invitrogen) at 4 °C for 15 min. Dynabeads were collected by magnet then washed 5 times with 500 µl Binding/Wash buffer. After final wash, buffer was aspirated and beads were incubated with 100 µl Elution buffer (300 mM imidazole, 50 mM Na-phosphate pH 8.0, 300 mM NaCl, 0.01% Tween-20) for 20 min, then beads were collected via magnet. The supernatant containing purified HIS_6_-Ssa1 was transferred to a fresh tube, 25 µl of 5× SDS-PAGE sample buffer was added and the sample was denatured by boiling for 5 min at 95 °C. 10 µl of sample was analyzed by SDS-PAGE. To isolate HIS_6_-tagged Hsp82, SKY4635 expressing HIS_6_-Hsp82 as the sole Hsp90 isoform in the cell [4] were grown and processed identically to the SKY4364 cells as above.

## 2 LC–MS/MS data acquisition

### 2.1 Trypsin digestion of samples from SDS-PAGE gels

Gel lanes to be analyzed were excised from 4% to 12% MOPS buffer SDS-PAGE gels by sterile razor blade and divided into 8 sections with the following molecular weight ranges: 300–150 kDa, 150–110 kDa, 110–80 kDa, 80–75 kDa, 75–60 kDa, 60–52 kDa, 52–38 kDa and 38–24 kDa. These were then chopped into ~1 mm^3^ pieces. Each section was washed in dH_2_O and destained using 100 mM NH_4_HCO_3_ pH 7.5 in 50% acetonitrile. A reduction step was performed by addition of 100 μl 50 mM NH_4_HCO_3_ pH 7.5 and 10 μl of 10 mM Tris(2-carboxyethyl)phosphine–HCl at 37 °C for 30 min. The proteins were alkylated by adding 100 μl of 50 mM iodoacetamide and allowed to react in the dark at 20 °C for 30 min. Gel sections were washed in water, then acetonitrile, and vacuum dried. Trypsin digestion was carried out overnight at 37 °C with 1:50 enzyme–protein ratio of sequencing grade-modified trypsin (Promega) in 50 mM NH_4_HCO_3_ pH 7.5, and 20 mM CaCl_2_. Peptides were extracted with 5% formic acid and vacuum dried.

### 2.2 Isotopic labeling

Peptide digests were reconstituted with 60 µl of Tris–HCl buffer solution (10 mM of Tris–HCl, 150 mM NaCl, 20 mM CaCl_2_, pH 7.6), then split into two vials with 30 µl each (^16^O vial and ^18^O vial) and vacuum dried. In a separate vial, 30 µl of Mag-Trypsin beads (Clontech) was washed 5 times with 500 µl of Tris–HCl buffer solution, then vacuum dried. 30 µl of either ^16^O H_2_O or 97% ^18^O H_2_O (Cambridge Isotopes) was added to the respective ^16^O or ^18^O vials and vortexed for 20 min to reconstitute the peptide mixture, which was then added to the prepared Mag-Trypsin bead vial and allowed to exchange overnight at 37 °C. After ^18^O exchange, the solution was removed and any free trypsin in solution was inactivated with 1 mM PMSF for 30 min at 4 °C. For each sample the +/−MMS digests were combined 1:1 as follows: Forward (FWD) Sample Set: (–MMS) ^16^O: (+MMS) ^18^O and Reversed (REV) Sample Set: (+MMS) ^16^O: (–MMS) ^18^O, dried and stored at −80 °C until analysis. Three biological replicate experiments were performed per sample.

### 2.3 HPLC for mass spectrometry

All samples were re-suspended in Burdick & Jackson HPLC-grade water containing 0.2% formic acid (Fluka), 0.1% TFA (Pierce), and 0.002% Zwittergent 3–16 (Calbiochem, a sulfobetaine detergent that contributes the following distinct peaks at the end of chromatograms: MH^+^ at 392, and in-source dimer [2M+H^+^] at 783, and some minor impurities of Zwittergent 3–12 seen as MH^+^ at 336). The peptide samples were loaded to a 0.25 μl C_8_ OptiPak trapping cartridge custom-packed with Michrom Magic (Optimize Technologies) C8, washed, then switched in-line with a 20 cm by 75 μm C_18_ packed spray tip nano column packed with Michrom Magic C18AQ, for a 2-step gradient. Mobile phase A was water/acetonitrile/formic acid (98/2/0.2) and mobile phase B was acetonitrile/isopropanol/water/formic acid (80/10/10/0.2). Using a flow rate of 350 nl/min, a 90 min, 2-step LC gradient was run from 5% B to 50% B in 60 min, followed by 50–95% B over the next 10 min, hold 10 min at 95% B, back to starting conditions and re-equilibrated.

### 2.4 LC–MS/MS analysis

The samples were analyzed via electrospray tandem mass spectrometry (LC–MS/MS) on a Thermo LTQ Orbitrap XL, using a 60,000 RP survey scan, *m*/*z* 375–1950, with lockmasses, followed by 10 LTQ CAD scans on doubly and triply charged-only precursors between 375 Da and 1500 Da. Ions selected for MS/MS were placed on an exclusion list for 60 s.

## 3 LC–MS/MS data analysis, statistical analysis and visualization

Data were analyzed and filtered on MaxQuant version 1.2.2 [5] (20 ppm error tolerance) with a FDR setting of 1% against the SPROT Yeast database and at a cutoff of at least 2 peptides seen to assign quantitation ratio. The exact MaxQuant settings used can be found in attached document. Each experiment was normalized to the ratio of the bait protein, i.e. SSA1 files using SSA1 ratio and HSP82 files normalized using HSP82 ratio. This produced a list of interactors and their respective quantitated changes upon DNA damage. Proteins were removed from the file if they were labeled as “Contaminants”, “Reverse” or “Only identified by site”. Three biological replicates were performed, with each biological replicate split into technical replicates (^18^O forward (FWD) labeling and ^18^O reverse (REV) labeling). A protein was considered identified if detected in at least three of the six replicates.

Statistical analysis was performed using the R statistical package (http://www.r-project.org/). Proteins with three out of six observations within each group (SSA1 and HSP82) were retained. Missing values were imputed using row mean imputation. *Z*-score normalization was performed on the log of all protein ratios. An ANOVA test was then performed to identify proteins that indicate significant variability (*P*-value<0.05) between biological replicates within each group. These were removed from consideration. The full data obtained was uploaded to the PRIDE repository and can now be found under reference number PXD001284.
